# Similar Impact of CD8+ T Cell Responses on Early Virus Dynamics during SIV Infections of Rhesus Macaques and Sooty Mangabeys

**DOI:** 10.1371/journal.pcbi.1000901

**Published:** 2010-08-26

**Authors:** Roger D. Kouyos, Shari N. Gordon, Silvija I. Staprans, Guido Silvestri, Roland R. Regoes

**Affiliations:** 1 Institute of Integrative Biology, ETH Zurich, Zurich, Switzerland; 2 School of Medicine, University of Pennsylvania, Philadelphia, Pennsylvania, United States of America; 3 Emory Vaccine Center, Atlanta, Georgia, United States of America; 4 Merck, Philadelphia, Pennsylvania, United States of America; Utrecht University, Netherlands

## Abstract

Despite comparable levels of virus replication, simian immunodeficiency viruses (SIV) infection is non-pathogenic in natural hosts, such as sooty mangabeys (SM), whereas it is pathogenic in non-natural hosts, such as rhesus macaques (RM). Comparative studies of pathogenic and non-pathogenic SIV infection can thus shed light on the role of specific factors in SIV pathogenesis. Here, we determine the impact of target-cell limitation, CD8+ T cells, and Natural Killer (NK) cells on virus replication in the early SIV infection. To this end, we fit previously published data of experimental SIV infections in SMs and RMs with mathematical models incorporating these factors and assess to what extent the inclusion of individual factors determines the quality of the fits. We find that for both rhesus macaques and sooty mangabeys, target-cell limitation alone cannot explain the control of early virus replication, whereas including CD8+ T cells into the models significantly improves the fits. By contrast, including NK cells does only significantly improve the fits in SMs. These findings have important implications for our understanding of SIV pathogenesis as they suggest that the level of early CD8+ T cell responses is not the key difference between pathogenic and non-pathogenic SIV infection.

## Author Summary

Simian immunodeficiency viruses (SIV) are typically non-pathogenic in their natural hosts. However, if the same virus infects a non-natural host it often leads to AIDS-like symptoms. Therefore, comparing SIV infections in these two types of host might help explain the pathogenesis of SIV in non-natural hosts and thereby also that of HIV. We combined mathematical modeling with data on the levels of virus and immune cells early in infection, and compared both non-pathogenic SIV infections of sooty mangabeys and pathogenic SIV infection of rhesus macaques with respect to how the virus grows in them and to what extent it is controlled by the immune system. We found that the impact of the immune system on early virus replication is remarkably similar in both species. In particular, for both species virus replication can only be explained by the effect of CD8+ T cells. These findings have important implications for our understanding of SIV pathogenesis as they suggest that the impact of the early immune responses is not the key difference between pathogenic and non-pathogenic SIV infection.

## Introduction

The simian immunodeficiency virus (SIV) occurs as a natural infection in several Old-world monkey species, such as sooty mangabeys (SM) or African green monkeys [1], [2]. In striking contrast to HIV infection of humans, SIV infection does not cause disease in natural hosts. The levels of virus replication, however, are similarly high in natural hosts and non-natural hosts such as rhesus macaques (RM), in which SIV causes AIDS-like symptoms. Comparative studies of SIV infection in natural and non-natural hosts provide the opportunity to investigate the interaction between the virus and the host immune system in pathogenic and non-pathogenic infection. Such a comparison might shed light on the mechanisms of disease progression in pathogenic SIV and by extrapolation on HIV.

Although natural and non-natural hosts allow similar levels of virus replication, there are interesting immunological differences: SMs do not exhibit the increased CD4+ T cell turnover and the generalized immune activation that is characteristic for the SIV infection of RMs or HIV-infection in humans [3], [4]. Thus, virus load alone cannot be the key to understanding pathogenesis. Silvestri and Feinberg [5] interpreted these findings in favor of the hypothesis that HIV disease progression is a result of generalized immune activation rather than of the destruction of CD4+ T cells by the virus alone. This view of HIV pathogenesis is a derivative of the immuno-pathological hypothesis [6]. Because primary HIV infection is a period critical for the future immune responses' capability of controlling the infection [7], [8], the potential differences between pathogenic and non-pathogenic SIV infection are likely to manifest themselves early in infection.

In both RMs and SMs, the early SIV infection is divided into three phases. The first phase is characterized by a sharp increase of virus load soon after infection. The second phase describes the decline of virus load that follows the initial peak viremia. The third phase finally describes the largely stable equilibrium virus load that eventually establishes after the decline. This stable virus load is also referred to as the viral set point. The characteristic pattern of virus load in primary SIV infection can be explained either through the delayed action of cellular immunity [9], [10] or through target cell limitation [11] or both. Note that in this context the term target-cell limitation refers to the hypothesis that the level of target cells on its own can explain the early virus-load dynamics [11]. Regoes et al. [9] investigated these hypotheses by fitting mathematical models to viral loads of SIVmac239-infected RMs that exhibited either normal or experimentally impaired cellular immunity as a result of co-stimulatory blockade. This analysis showed that target-cell limitation can explain the virus-load dynamics in the animals with impaired cellular immunity but not in those with a normal immune response. In the latter case, the models could explain the virus-loads only if cellular immunity is also taken into account. These results imply that target-cell limitation alone cannot explain the level of virus replication during primary SIVmac239 infection of RMs and thus suggest a role for cellular immunity in determining the post-peak decline of viremia.

In this article, we use the method of Regoes et al. [9] to analyze the early virus dynamics in non-pathogenic SIV infection of sooty mangabeys (SM). In particular, we sought to determine the roles that target-cell limitation, CD8+ T cell responses and NK cells play in primary infection of SMs, and to compare the impact of these factors with that in SIV-infected RMs. To this end we fit the measurements of virus load with population-dynamic models that differ as to whether they take factors such as cellular immunity or NK cells into account. Comparing the goodness of fit of these models, we can then evaluate the role of these factors in the primary infection of pathogenic and non-pathogenic SIV.

## Results

**Figure 1:**
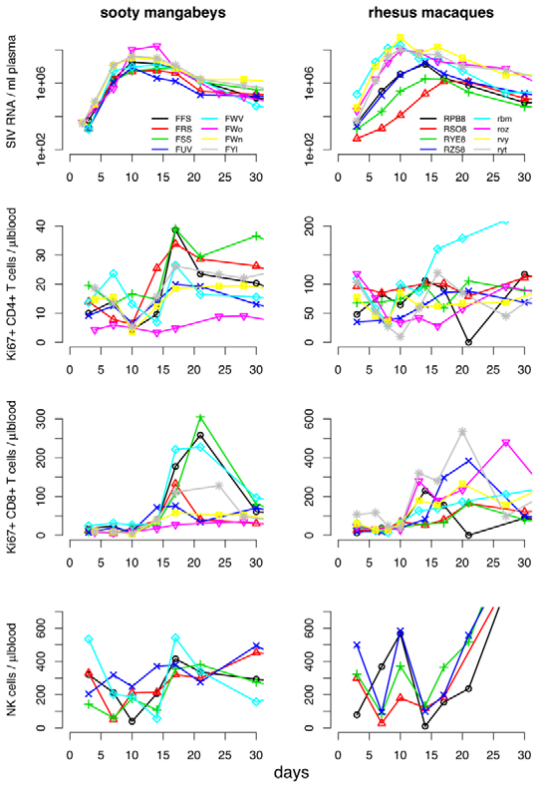
Measurements of virus loads and cell counts. Measurements of virus load (first row), proliferating CD4+ T-cells (second row), proliferating CD8+ T-cells (third row), and NK cells (fourth row) in sooty mangabeys and rhesus macaques.

**Figure 2:**
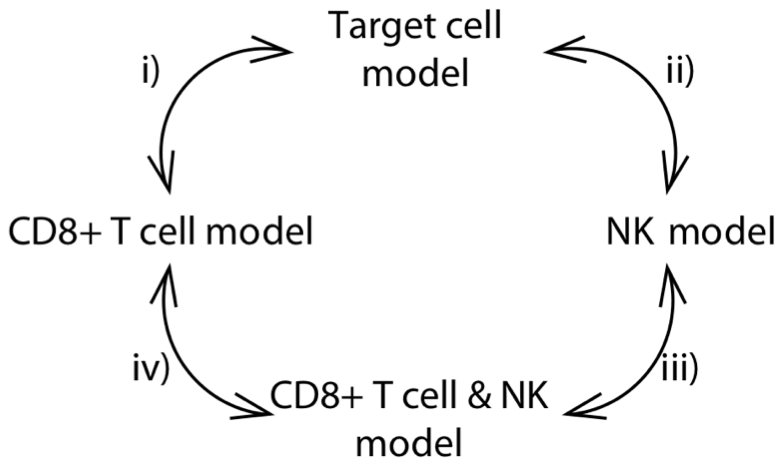
Illustration of the model comparisons. Comparisons i) and iii) test whether taking CD8+ T cells into account improves the fits of the target-cell model and the NK model, respectively. Comparisons ii) and iv) test whether taking NK cells into account improves the fits of the target-cell model and the CD8+ T cell model respectively.

We used previously published data of experimental SIV infections (see Figure 1) to assess the relative importance of target-cell limitation, CD8+ T cells, and NK cells for controlling virus replication in primary SIV infection. To this end, we determined to what extent the ability of mathematical models to fit the early virus dynamics depends on the inclusion of these factors (see Figure 2). We start by showing that CD8+ T cells in combination with target-cells, but not target cells on their own, can explain the early SIV dynamics in RMs. Then we show that cellular immunity has a similar effect in early SIV replication of both RMs and SMs. Finally, we argue that NK cells only have an impact on the early replication in SMs.

The target-cell model aims to explain the virus-load dynamics through target-cell limitation only (equation 1), whereas the CD8+ T cell model takes both target-cell limitation and cellular immunity into account (equation 2). We use the density of proliferating CD4+ T-cells and of proliferating CD8+ T-cells as proxies for the size of the target cell population and for the strength of the specific cellular immunity. Comparing these two models assesses the relative role of target-cell limitation and cellular immunity in controlling the virus load: A good fit of the target-cell model and an only insignificant improvement in the CD8+ T cell model, would suggest that the virus load is mainly controlled by target-cell limitation. On the contrary, a bad fit of the target-cell model and a significant improvement in the CD8+ T cell model, would support the view that cellular immunity plays an important role.

### Target-cell limitation does not explain virus control in rhesus macaques

**Figure 3:**
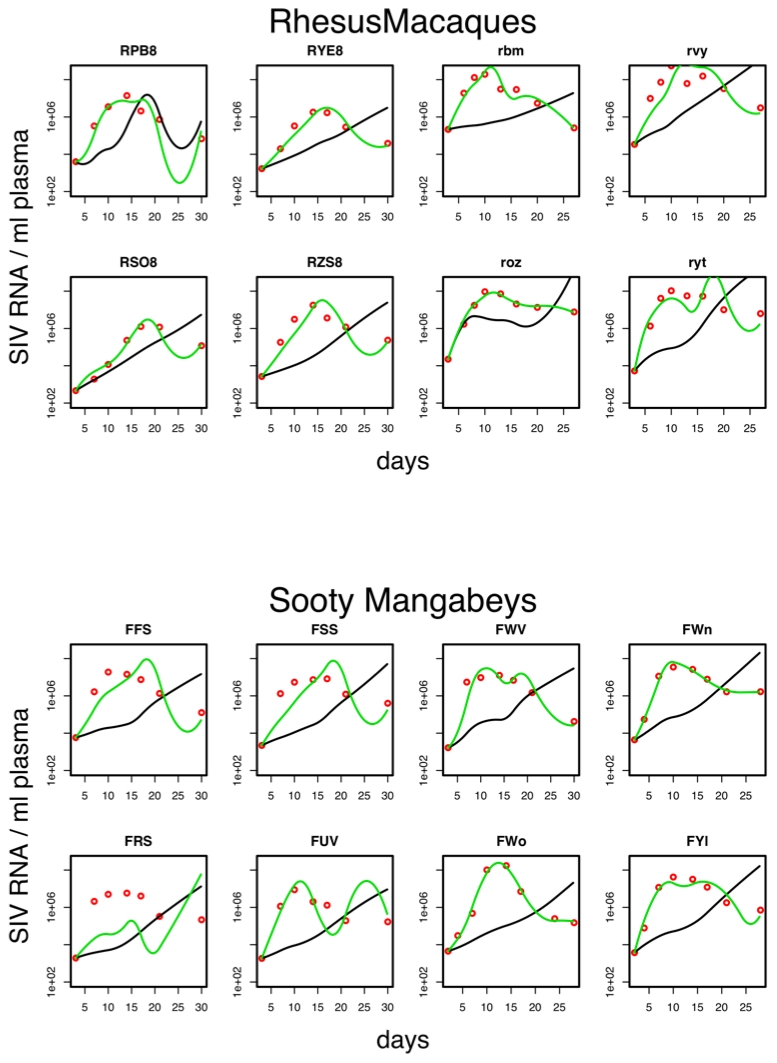
Best fits of virus load data. Best fits by the target-cell model (black lines) and the CD8+ T cell model (green lines) of the virus load measurements (red dots) of sooty mangabeys and rhesus macaques.

The analysis of the RM data reconfirms the results of Regoes et al. [9] in an extended dataset. In particular, we find that target-cell limitation alone cannot explain the virus dynamics. For all animals except one (animal *RPB*8), the best fit of the target-cell model predicts a steadily increasing virus load (black lines in Figure 3), i.e. the fit fails to explain the characteristic peak and the subsequent post-peak decline exhibited by the data. Moreover, the quality of the fit is poor even for the animal for which the target-cell model can predict a viral load decrease. Adding specific cellular immunity to the target-cell model does significantly improve the fit for RMs (*F*-test, *p* = 2.8×10^−18^). Importantly, the CD8+ T cell model can explain the characteristic post-peak decline of the viral load (green lines in Figure 3).

### Target-cell limitation does not explain virus control in sooty mangabeys

The results of our analysis of the data from SIV infection of SMs are strikingly similar to those obtained for the rhesus macaques: The target-cell model fails to explain the virus dynamics for all eight animals (Figure 3), whereas the CD8+ T cell model provides a significantly better fit (*F*-test, *p* = 1.3×10^−11^), which can reproduce the qualitative patterns of the virus dynamics. The only exception is the animal *FSS*, for which both the target-cell and the CD8+ T cell model produce poor fits. The poor quality of these fits might be due to the fact that this animal exhibits a comparatively early increase of target-cell number and a comparatively late increase of CD8+ T-cell number (see Figure 1). The similarity of the results in SMs and RMs suggests that the relative importance of specific cellular immunity and target-cell limitation during early infection is comparable in pathogenic and non-pathogenic SIV hosts. In both cases, the temporal dependence of the viral load can only be explained if CD8+ T cells are taken into account.

Table 1 shows the best-fit estimates and the confidence intervals for the parameters of the CD8+ T cell model. The parameters *r* and *k* quantify the per-cell impact of target-cells and CD8+ T-cells on the viral replication rate (see equation 2). Both parameters are on average higher for sooty mangabeys: *r* roughly by a factor 6 and *k* by a factor 3. Furthermore, the intrinsic death rates of infected cells, *a*, were estimated to be 0 for most animals. This suggests that, for both SMs and RMs, most deaths of infected cells are caused by cellular immunity (see [9]).

**Table 1:** Parameter estimates.

Animal	a(95% CI)	r (95% CI)	k (95% CI)	SSQ1	SSQ2
*Sooty Mangabeys*					
FFS	0 (0,8)	0.1 (0.077,0.92)	0.017 (0.012,0.09)	3.3	29
FRS	0 (0,2.8)	0.08 (0.0011,0.6)	0.033 (0.0021,0.19)	25	30
FSS	0 (0,8.8)	0.055 (0.046,0.67)	0.011 (0.0092,0.099)	4.2	27
FUV	0 (0,3.6)	0.21 (0.19,0.62)	0.066 (0.056,0.12)	2.3	23
FWV	1.8 (0,150)	0.19 (0.094,7.9)	0.011 (0.0023,0.19)	1.6	30
FWo	0.2 (0,2.2)	0.45 (0.35,0.78)	0.11 (0.094,0.13)	0.21	33
FWn	0.71 (0.26,2.2)	0.19 (0.14,0.3)	0.055 (0.041,0.063)	0.15	25
FYl	1.1 (0,1.9)	0.16 (0.084,0.25)	0.03 (0.021,0.047)	0.69	30
*Rhesus Macaques*					
RPB8	2.6 (0,5.3)	0.059 (0.019,0.1)	0.017 (0.0088,0.024)	1.9	12
RSO8	4.2 (0,24)	0.055 (0.0021,0.28)	0.0085 (0.0035,0.017)	0.3	6.3
RYE8	0 (0,13)	0.016 (0.015,0.23)	0.015 (0.014,0.12)	0.41	12
RZS8	0 (0,0)	0.025 (0.021,0.038)	0.009 (0.007,0.02)	1.6	22
rbm	0.017 (0,2)	0.034 (0.031,0.082)	0.037 (0.034,0.07)	0.67	25
roz	0 (0,0.28)	0.018 (0.017,0.023)	0.0043 (0.0032,0.0047)	0.13	11
rvy	0 (0,29)	0.028 (0.023,0.53)	0.011 (0.0089,0.029)	3.8	30
ryt	0 (0,0.22)	0.043 (0.035,0.056)	0.011 (0.008,0.015)	2.2	34

Parameter estimates for the best fit with the CD8+ T cell model (*a*, *r*, and *k*). Residual sum of squares of the CD8+ T cell model (*SSQ1*) and the target-cell model (*SSQ2*). The confidence intervals (CI) are derived from bootstrap estimates for 1000 re-sampled datasets. The re-sampling involved choosing time-points with replacement. Rates are given in units of *days* ^−1^.

### Impact of NK-cells in rhesus macaques and sooty mangabeys

The NK cell model and the CD8+ T cell & NK model are obtained from the target-cell and the CD8+ T cell model by adding NK cell number as an explanatory variable. We consider the fits of these extended models for two reasons: First, to test whether the above results are robust against adding NK cells to the model and, second, to investigate the role of an important effector mechanism of the innate immune system during primary SIV infection. In total, four types of statistical comparisons were performed (see Figure 2): Comparison i) between the target-cell model and the CD8+ T cell model is the one discussed above. Comparison ii) between the target-cell model and the NK model evaluates whether adding NK cells to target-cell limitation improves significantly the quality of fit. Comparison iii) between the NK model and the CD8+ T cell-NK model evaluates whether taking cellular immunity into account improves the fit of the NK model. Finally, comparison iv) assesses whether NK-cell number does significantly improve the fit of the CD8+ T cell model.

NK cell counts were available for 8 SMs (*FWo*, *FYl*, *FWn*, *FFS*, *FRS*, *FSS*, *FUV*, *FWV*) and 4 RMs (*RPB8*, *RSO8*, *RYE8*, *RZS8*). If the number of all NK cells is used as a proxy of NK cell activity, extending the target cell based model by NK cells (comparison ii) does improve the model fits significantly only for SM but not for RM (*F*-test, *p* = 0.016 and p = 0.24 for SM and RM, respectively). Extending the CD8+ T cell model by NK cells failed for both species to improve the model fits significantly (*F*-test, *p* = 0.98 and p = 0.33 for SM and RM, respectively). In contrast, extending the NK model by CD8+ T cells improves the fit significantly (*F*-test, *p* = 2.4×10^−5^ and p = 5.7×10^−4^ for RM and SM, respectively). If the number of proliferating NK cells is used as a proxy of NK cell activity, including NK cells again significantly improves the target-cell based model only for SM (*F*-test, *p* = 1.3×10^−5^ and p = 0.33 for SM and RM, respectively). In addition, including NK cell activity via this proxy also improves the CD8+ T cell model for SM (*F*-test, *p* = 0.00013 and p = 0.97 for SM and RM, respectively). These results suggest that NK cells play a role in the early infection of SM but not of RM.

## Discussion

The role of cellular immunity in early SIV/HIV infection has been a debated topic since the suggestion of Phillips [11] that early virus replication might be controlled by target-cell limitation. Several lines of evidence suggest however that cellular immunity is an important force for the control of early SIV replication. First, the post-peak decline of virus load coincides temporally with the rise of CTLs [12](although this is also consistent with the alternative explanation of [11]). Second, [10] have shown that the post-peak decline of virus-load is significantly weakened if CD8+ T-cells are depleted. Third, the ubiquitous selection for mutants that escape CTL response [13] also suggests an important role of cellular immunity. Fourth, it has been shown that the patients' ability to control HIV depends strongly on the alleles at the HLA and KIR loci [14], which control the action of CD8 T cells and NK cells, respectively. More recently, some of the authors of this paper [9] have shown that mathematical models can explain the early virus dynamics if they take both target-cells and CD8+ T-cells into account, but not if they take only target cells into account. Our study extends this previous work by considering the impact of NK cells, important effectors of innate immunity. In addition to the extended analysis of the early viral dynamics in pathogenic SIV infection, we here compare our results to non-pathogenic SIV infection in sooty mangabeys (SMs). This comparison has important implications for our understanding of pathogenesis.

Our analysis confirms the earlier finding of [9] that target-cell limitation alone cannot explain the virus dynamics in RMs. We find that, in SIV-infected sooty mangabeys, target-cell limitation is equally unable to explain the viral load dynamics during early infection. In both species, our model can only explain the virus dynamics if it takes cellular immunity into account. This suggests that specific cellular immunity plays an important role in determining the dynamics of virus replication during early infection in both species. We, however, also found that a model, which assumes a constant viral replication rate, independent of target cells, was unable to fit the virus-load data of all animals consistently (results not shown). This implies that, although target cells alone cannot explain the virus-load dynamics, in particular the peak and the post-peak decline, temporal variation of target cells is nevertheless important. Overall, our results indicate that the relative impact of target-cell limitation and specific cellular immunity is similar in RMs and SMs.

These results give rise to testable predictions. If, for example, one would selectively deplete NK cells during primary infection, the pattern of virus load should be affected in SM, but not in RMs. In contrast, selective depletion of CD8+ T cells is predicted to lead to a loss of control of virus replication in both species. Of note, all depletion experiments performed using an anti-CD8 antibody depleted CD8+ T cells as well as NK cells because both cell types express CD8 [10]. In RMs, treatment with a costimulatory inhibitor, which prevented the development of SIV-specific cellular and humoral immunity and reduced target cell levels, gave rise to target cell limited virus replication [9]. The similarity between the factors governing virus replication predicts that an analogous treatment of SMs would also lead to target cell limitation.

Our conclusions about the role of cellular immunity and target-cell limitations are based on several assumptions. First, the virus loads and the immune-cell densities were measured in the blood, which is not the main compartment of SIV replication and lymphocytes. Our analysis, therefore, relies on the assumption that the measurements in the blood reflect the situation in the whole body. In this context, it has been suggested that target-cell depletion in the gut might play an important role in the early SIV infection [15], [16], [17], [18], [19], [20], [21], [22]. However, a recent study has shown that in SIV infections of both SM and RM, the target-cell depletion in the gut occurs too early to explain the peak in virus-load [23]. Second, our models consider only the primary phase of SIV infection. Therefore, our conclusion that cellular immunity does not differ in pathogenic and non-pathogenic SIV, does only apply to this phase. It might thus be that cellular immunity at later phases plays a very different role in RMs and SMs, as suggested by numerous comparative studies [2], [3], [24], [25]. As discussed in Regoes et al. [9], it is difficult to extend the approach used here to later phases of infection, because immune-escape and antibody responses would require considerably more complicated models. Last, we cannot exclude that different cell compartments or cell types play the role of target cells in the SIV infections of sooty mangabeys and rhesus macaques. Indeed, our model fits result in larger replication rate constants, r, for SM than for RM, which either suggests a better target cell utilization in SM, or is an indication that Ki67+ CD4+ T cells do not play the same roles in SM and RM. Such an effect could systematically bias our analysis if our proxy (i.e. proliferating CD4+ cells) would be representative for target cells in one species but not in the other. Finally, the *p* values of the model comparisons rely on the assumptions of normality and independence, which might be violated in our data. Especially, autocorrelation in the virus-load and cell-numbers, might potentially lead to an overestimate of the degrees of freedom and thereby to an underestimate of those *p-values*. However, it should be noted that independently of the statistical evaluation, the least-squares approach is a simple and intuitive method to fit dynamical models to data, and these fits clearly (Figure 3) show that for all animals except one RM (RPB8), the best fit of the target-cell-limitation model fails to predict a post-peak decrease in virus-load. This suggests that our results regarding the CTLs are robust against these (in principle valid) statistical concerns. By contrast, adding NK cells to the model leads to smaller improvements of the fits and therefore these findings may be more vulnerable to potential autocorrelations.

One important caveat mentioned in the previous section is the uncertainty as to whether the measured cell populations (e.g. Ki67+ CD4+ T-Cells, Ki67+ CD8+ T-Cells, NK cells) can be identified with populations performing a specific function (target cells, cytotoxic T cells, cytotoxic NK cells). This potential problem is substantially alleviated by the way these measurements are integrated into our model. Specifically, the quality of fit as measured by the residual sum of squares, is invariant with respect to a linear transformation of the variables. I.e. if we measure the cell population x but the active population is x′ = a x-b we will obtain the same quality of fit regardless of whether we incorporate x or x′ into our model. Therefore it does not matter whether only a fraction of the measured cells is active or whether a constant number of the measured cells is inactive. For practical reasons, however, it is important that the fraction of the active cells is not too small relative to the inactive cells, because then the noise in the latter is likely to overwhelm the signal in the former. This reasoning implies that the comparison of the quality of fit of the different models (Figure 2) is much more robust than the parameter estimates (Table 1): In principle, the first type of analysis (model comparison) still works, even if the linear transformation (relating measured cell populations to the cell-populations performing a specific function) is different for each animal. By contrast, the second type of analysis (parameter estimation) requires that this transformation is similar in the animals compared. For these reasons, we conclude that not too much weight should be given to the parameter estimates, as they rely much stronger on a good match between measured cell populations and the populations actually performing a certain function, while we can assert that the model comparison is robust. The fundamental robustness of the method also explains why [9] found qualitatively similar results with Ki67+ CD8+ T-cells and tetramer positive T-cells as markers for SIV-specific cellular immunity.

As SIV infection is pathogenic in rhesus macaques but non-pathogenic in sooty mangabeys, our results can be interpreted in the context of current theories of SIV pathogenesis, in particular with respect to reasons underlying the absence of disease progression in SIV-infected SMs. While an initial study suggested that acute SIV infection of SMs is characterized by limited to absent T cell activation [25], a number of more recent studies that included a more comprehensive sample collection have shown very clearly that SMs exhibit substantial T cell activation during acute SIV infection [24], [26], [27], [28]. However, in marked contrast with SIV-infected RMs, sooty mangabeys are able to rapidly and dramatically reduce the level of T cell activation during the early chronic infection (i.e., starting at day 30 post inoculation) [24], [26], [27], [28]. Although our model comparison did not directly test differences in the antigenicity of SIV between SM and RM, our results are more consistent with the latter observations and suggest that the divergent outcome of SIV infection in RMs and SMs is not caused by differences in CD8+ T-cell response during the early stages of infection.

## Methods

### Ethics statement

All the experiments on non-human primates from which these data are sampled have been approved by the Institutional Animal Care and Use Committee (IACUC). All these experiments have been described in previous publications.

### Data

The data analyzed in this article were generated in experimental infections of SMs infected with the viral strain SIV_smm_ and of rhesus macaques infected with the strains SIV_mac_ (animals *rbm*, *rvy*, *roz*, *ryt*) or SIV_smm_ (animals *RPB8*, *RSO8*, *RYE8*, *RZS8*, *RFT8*). A detailed description of the experiments can be found in Garber et al. [29], Gordon et al. [30], and Mandl et al. [4]. For the sake of comparability, we consider the same time-window as Regoes et al., i.e. a window ranging from day 0 (start of infection) to day 30. In one of the rhesus macaques (animal *RFT8*) no SIV infection could be established. This animal was therefore excluded from further analysis. In total, we consider 8 SMs (all infected with SIV_smm_) and 8 RMs (4 infected with SIV_mac_239 and 4 infected with SIV_smm_).

Figure 1 shows the measurements relevant for this study: the virus-load, the density of proliferating CD4+ T-cells, the density of proliferating CD8+ T-cells, and the density of NK cells. The fraction of proliferating CD4+ and CD8+ T cells was assessed by staining for the nuclear antigen Ki67, which is expressed by cycling cells. We consider the density of proliferating CD4+ T-cells as representative for the size of the target cell population and the density of proliferating CD8+ T-cells as a surrogate measure for the SIV-specific cellular immunity. We will therefore refer to the density of proliferating CD4+ T-cells and of proliferating CD8+ T-cells also as “target cells” and “cellular immunity”, according to the functional role we assume these populations to play. Data on the density of NK cells were only available for all sooty mangabeys and for 4 out of 8 rhesus macaques (*RPB8*, *RSO8*, *RYE8*, *RZS8*).

### Models

The data were analyzed by using population dynamic models, which describe the virus dynamics as a function of target cells, CD8+ T-cells, and NK cells. The models are fitted to the virus load. Hereby, the measurements of target cells, CD8+ T-cells, and NK cells were used as explanatory variables. Importantly, the model does not aim to explain the measurements of these cell populations, but considers them only as factors that might explain viral replication. A detailed account of this approach can be found in [9].

In order to assess the role of target-cell limitation and cellular immunity in early SIV infection, we compared the fits of two nested models, which describe the virus dynamics by taking into account either target cells only or target cells and specific cellular immunity. These models are referred to as the target-cell model and the CD8+ T cell model, respectively. Mathematically, these models read
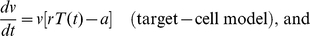
(1)

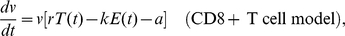
(2)where *v* is the virus load and *T(t)* and *E(t)* denote the number of proliferating CD4+ T-cells and of proliferating CD8+ T-cells, respectively. The parameters *r*, *a*, and *k* are chosen for each animal such that *T(t)* and *E(t)* give the best possible prediction of *v* (see below).

In order to test the impact of the non-adaptive immune system on our results, we extended the above models by adding NK cell number as an explaining factor. We incorporate the impact of NK cells by using two different proxies: either the total density of NK cells (characterized as CD3− CD20− CD16+ cells) or only the density of activated NK cells (i.e. Ki67+ NK cells). The second approach is identical to the one used of CD8+ and CD4+ T-cells. The first approach can be justified by the fact that, in contrast to CD8+ T-cells, NK cells do not recognize specific antigens. Thus, every NK cell can potentially inhibit virus replication by either killing infected cells or by IFN-gamma production [31], and their effect is most likely proportional to their level. We would like to emphasize that we do not assume that every NK cell is cytotoxic, or that every NK cell has anti-viral activity. We only assume that the impact of NK cells is proportional to their abundance (see discussion). The extensions of the target-cell model and the CD8+ T cell model are referred to as the NK-model and the CD8+ T cell & NK model. Mathematically these models read
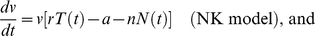
(3)

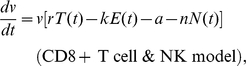
(4)where *N(t)* denotes the number of NK cells and the parameter *n* is chosen according to the best fit criterion.

### Fitting and statistics

We illustrate the fitting-procedure for the CD8+ T cell & NK model: First the differential equation of the model (4) can be integrated to
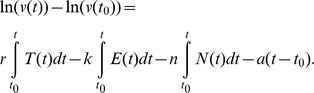
(5)If *t_0_…t_k_* , denote the time points for which measurements of *v* are available then the parameters *r*, *k*, *a* and *n* are chosen such that the residual sum of squares
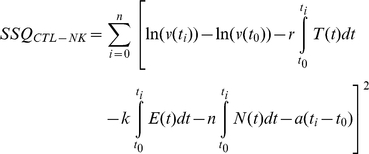
(6)is minimized. The integrals in the sum are computed from the measurements of the cell numbers *T* ,*E*, and *N* by first interpolating these measurements by a piecewise linear function, resulting in the functions *T(t)*, *E(t)*, and *N(t)*, and then integrating these interpolating functions. As expression (5) is linear in the parameters *r*, *k*, *a* and *n*, the best fit can be found using a standard linear-model solver such as the *lm()* routine of the *R* language [32]. Biologically, the parameters *r*, *k*, *a* and *n* must be larger than or equal to 0. If the best fit of (5) does not fulfill these conditions, one or several of the parameters *r*, *k*, *a* and *n* is set to 0 and the fitting procedure is repeated with these reduced functions. From all the “reduced fits”, that one is chosen, which yields the minimal sum of squares while fulfilling the biological conditions.

The fits for the target-cell, the CD8+ T cell, and the NK model are obtained in a similar way as for the CTL-NK model. In formula (5) the parameters that do not occur in the differential equation of the model (i.e. equation 1, 2, or 3 for the target-cell, CD8+ T cell, and NK model respectively) are set to 0 and the remaining parameters are chosen such that the corresponding sum of squares (*SSQ_target-cell_*, *SSQ_CD8+ T cell_*, and *SSQ_NK_*) is minimized.

We can statistically compare two of the above models, for instance model 1 and model 2, if they are nested, i.e. if model 1 results from model 2 by setting one of the parameters to 0. In such cases, model 2 will always provide a better fit than model 1, because model 1 is included as a special case in model 2. Whether this improvement in the quality of fit is significant can then be assessed by performing an *F*-test. The corresponding test statistic is
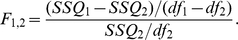



Here *SSQ_i_* denotes the residual sum of squares of the model i, and *df_i_* refers to the corresponding degrees of freedom. The *p* value that corresponds to the value of *F* is then calculated from the Fisher Distribution with degrees of freedom *df_1_-df_2_* and *df_2_*, i.e *F(df_1_-df_2_, df_2_)*. This comparison between models can be made either for each animal individually, or, as we mostly do in this article, for all animals of a species taken together. In the latter case, the residual sum of squares obtained by fitting the models to each animal and their corresponding degrees of freedom have to be summed to perform the *F*-test.

Figure 2 illustrates the statistical comparisons that are made in this article. The most important of these comparisons is the one between the target-cell model and the CD8+ T cell model (comparison i in Figure 2), which assesses the relative importance of target cells and specific cellular immunity for explaining the virus-load dynamics. If NK-cell counts are available, one can ask in addition whether taking NK cells into account improves the fit of the target-cell model (comparison ii), whether taking specific cellular immunity into account improves the fit of the NK model (comparison iii), and whether taking NK cells into account improves the fit of the CD8+ T cell model (comparison iv).
